# Design of Political Online Teaching Based on Artificial Speech Recognition and Deep Learning

**DOI:** 10.1155/2022/3112092

**Published:** 2022-10-06

**Authors:** Xiajin Chen

**Affiliations:** Yiwu Industrial Commercial College, Yiwu, Zhejiang 322000, China

## Abstract

With the emergence of the information age, computers have entered the homes of ordinary people and have become essential daily appliances for people. The integration of people and computers has become more popular and in-depth. Based on this situation, how to make computers and humans communicate intelligently and make human-computer interaction more convenient and practical is the main issue of scientific research and discussion on computers. Language recognition is widely used in industry, the economy, commerce, tourism, style, and sports. The BP neural network based on deep learning has supercomputing functions and is very suitable for speech recognition, which promotes the promotion and application of speech recognition technology in many fields. Different types of applications and diverse scenarios enable users to experience different personal experiences. This paper examines the use of application software for ideological and political theory courses in colleges and universities and the application of application software for ideological and political theory courses in colleges and universities in the management of ideological and political theory courses. Analyzed the use of apps in the management of ideological and political theory courses in colleges and universities, analyzed the possibilities and challenges that this brought to the management of ideological and political theory courses in colleges and universities based on the app, and put forward the necessary countermeasures. Including the development and design of ideological and political theory applications, combining deep learning and CTC algorithms to build acoustic models, using server-client interaction, designing an ideological and political theory course app based on speech recognition and deep learning, and forming an offline speech recognition system software platform, the app provides a certain reference for improving the skills of teaching managers.

## 1. Introduction

With the development of Internet technology and the unremitting efforts of manufacturers of mobile devices in various countries in the world, wireless mobile terminal devices have become very popular in people's daily work, production, and lives [1]. With the drastic reduction of communication costs, the rapid increase of network speeds, and the full coverage of wireless networks, all these increasingly perfect conditions have accelerated the rapid promotion and popularization of mobile terminals such as smartphones and tablet computers [2]. The proportion of Internet equipment used by everyone has been rapidly increasing and has gradually become the most important mobile terminal equipment used by people, gradually replacing the use of computers and notebooks in people's lives [3]. Therefore, it is no exaggeration to say that smartphones have been closely linked with our work, production, and life and have become an indispensable item in our daily work and life, and even an essential thing in our daily lives. In the current period, with the deepening of research, the exploration of the teaching and management of ideological and political theory courses has gradually deepened. Various applications in smartphones are very convenient and simple, with high efficiency and low cost. They can fully meet the requirements of students to learn anytime, anywhere, and are favored by students [4]. This is the development trend of learning methods in the future. At this stage, the number of mobile Internet users in my country is very large. Due to the rapid improvement of the educational information industry and the strong growth of user demand, apps as an application are indispensable in education and teaching [5]. It can not only promote the integration of educational theory and information technology but also strengthen the dissemination of educational ideas and scientific theories.

With the emergence of the information age, computers have entered the homes of ordinary people and have become essential daily appliances for people. The integration of people and computers has become more popular and in-depth [6]. Based on this situation, how to make computers and humans communicate intelligently and make human-computer interaction more convenient and practical is the main issue of scientific research and discussion on computers. Language recognition is widely used in industry, economy, commerce, tourism, style, and sports [7]. The BP neural network based on deep learning has super-computing functions and is very suitable for speech recognition, which promotes the promotion and application of speech recognition technology in many fields. After the emergence of new media, while breaking with the original advantages of the old media, the content and methods of teaching and management of ideological and political theory courses have been updated, which stimulated students' interest in learning, creativity, and teachers' enthusiasm [8]Better management efficiency provides new methods for ideological and political theory education. However, the application of apps is extremely rich and complex, which not only provides new methods for ideological and political education management but also brings great challenges [9]. The requirements for teachers and students are higher, and its freedom, diversity, and rapid dissemination also pose challenges and puts forward higher requirements for teachers' abilities. Therefore, the need to combine some modern computer science and technology has become the focus of this article.

## 2. Related Work

For a long time, speech recognition has mainly focused on speech machines and natural language processing, as well as the sound signal itself. People obtain a lot of outside information through human-computer interaction. About 76% of people's daily communication is conducted through speech. Automatic language recognition technology is also called speech recognition technology, which is the technology responsible for converting speech into text [10]. This includes technology and knowledge of multiple disciplines. Automatic speech recognition technology performs speech recognition in human-computer interaction. Automatic speech recognition technology is committed to completing the smooth communication between humans and machines. The world's first speech recognition system was first researched and developed by American scientists. At first, it could only recognize simple numbers, but it provided new ideas for the development and research of automatic speech recognition technology. Compared with the original text interaction method, the method of human-computer interaction using speech recognition technology is more suitable for people's habit of communicating through language [11]. In addition, voice interaction will be smoother, more efficient, and have fewer errors. Voice recognition technology is mainly used in voice management systems, intelligent dialogue inquiry systems, dictation, transcription systems, and situational dialogue systems. In the beginning, researchers used pattern engineering methods to build a speech recognition system, but the results were minimal.

After the emergence of deep learning, speech recognition technology has gradually reached a modern level on a large scale [12]. This research study uses a deep neural network, and the research proves that, compared with previous research results, the recognition error rate has been significantly reduced by 40%. The literature records a deep-faith network that pre-trains deep neural networks layer by layer through a limited Boltzmann machine, which greatly reduces the difficulty of deep neural networks and significantly improves the modeling level of neural networks. The study of neural networks has triggered a wave of deep learning. In depth neural network and hidden Markov mixture model, depth neural network is used to replace Gaussian mixture model, and speech signal model is established. Great progress has been made in speech recognition effect. The reference records a sequence discrimination technique that makes deep neural network training directly determine the level of recognition frequency, greatly reducing the huge error in recognition and becoming a standard part of subsequent deep neural network training. Literature established a highly consistent speech-recognition neural network [13]. The literature accurately simulates the correlation between the different dimensions of the speech frequency spectrum function, shows a high level of anti-noise, and greatly improves the noise reduction ability of the deep neural network. Researchers used long-term and short-term memory models in speech recognition. People use long-term historical framework information to make more precise decisions and achieve better results than prospective neural networks. Researchers used short-term classification termination technology in speech recognition, which severely weakened the role of HMM (a technology that has been used for nearly half a century) and significantly improved the performance of continuous numbering and other items [14]. A codec-based speech recognition scheme is used. The input of this end-to-end model is the speech spectrum function, and the output is direct words. The original acoustic model and language model are interwoven into a deep neural network for common modeling, almost completely destroying the traditional speech recognition framework.

Digital recognition technology is one of the key technologies for mobile application development. The term “mobile learning” refers to a type of learning that can be performed at any time and any place using mobile computer equipment. Mobile computer equipment used for mobile learning requires a certain ability to display learning content and provide teachers with communication between students [15]. Technical features of the new system include intelligence and digitization, digital transformation and coding, and new functions beyond the media. The literature explains the important role of mobile media devices. From the birth of human beings to the birth of mobile phones, these two functions have been separated [16]. The development of science and technology has merged these two mutually independent parts. The record pointed out that the emergence and use of the Internet has improved the level of cultural thought and educational technology, and the idea of educational management has been added to cultural and technical learning to guide and inspire students. Media literacy education is no longer a violation of students. The education of media experience is no longer regarded as a means of discrimination or an observation of ideology, and pointed out that the future of media education will be different [17]. It has the characteristics of socialization. It can improve its own quality through traditional school education. Participate in media life to achieve social media education and improve students' social participation. The positive and negative effects of the Internet era on college education are used as a basis to study on how to improve and perfect college ideological and political theory education to cope with the changes of the times.

## 3. Research on Speech Recognition Control System Based on Deep Learning

### 3.1. Deep Learning Model and Algorithm Application

**Figure 1:**
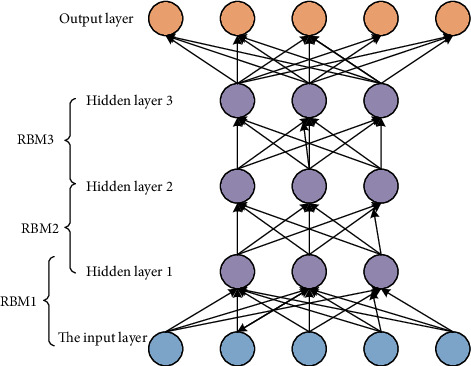
Deep belief network structure model.

The original neural network is a decision model, and the deep-faith network is a hybrid model, which combines a generative model and a decision model, as shown in Figure 1. Therefore, the network can realize the common probability arrangement of observation data and tags, which is convenient for estimating the former probability and the latter probability. In the deep trust network model, the middle hidden layer is mainly composed of limited Boltzmann machines.

The neurons in the speech recognition system present a Gaussian distribution, so neuron V is a hidden neuron *h*. Under this condition, the energy function is

(1) $$\begin{array}{c} E\left(v,h\left|\theta \right.\right)=\overset{n}{\underset{i=1}{\sum }}\frac{{\left(v_{i}-a_{i}\right)}^{2}}{2\sigma_{i}^{2}}-\overset{m}{\underset{j=1}{\sum }}b_{j}h_{j}-\overset{n}{\underset{i=1}{\sum }}\overset{m}{\underset{j=1}{\sum }}\frac{v_{i}}{\sigma_{i}}W_{ij}h_{j}\text{.} \end{array}$$

In the energy function, the conditional probability that can be achieved is

(2) $$\begin{array}{c} P\left(v_{i}=1\left|h\right.\right)=N\left(a_{i}+\underset{i}{\sum }W_{ij}h_{j},\sigma_{i}^{2}\right)\text{,}\\ P\left(h_{j}=1\left|v\right.\right)=S\left(b_{j}+\underset{j}{\sum }\frac{v_{i}}{\sigma }W_{ij}\right)\text{.} \end{array}$$

In order for the contrast divergence algorithm to be quickly applied, the input data of visual data is usually normalized into multiple islands with an average value of 0 and a variance of 1. The energy function and conditional probability are changed to

(3) $$\begin{array}{c} E\left(v,h\left|\theta \right.\right)=\overset{n}{\underset{i=1}{\sum }}\frac{{\left(v_{i}-a_{i}\right)}^{2}}{2\sigma_{i}^{2}}-\overset{m}{\underset{j=1}{\sum }}b_{j}h_{j}-\overset{n}{\underset{i=1}{\sum }}\overset{m}{\underset{j=1}{\sum }}\frac{v_{i}}{\sigma_{i}}W_{ij}h_{j}v_{i}\text{,}\\ P\left(h_{j}=1\left|v\right.\right)=S\left(b_{j}+\underset{j}{\sum }W_{ij}\right)\text{.} \end{array}$$

**Figure 2:**
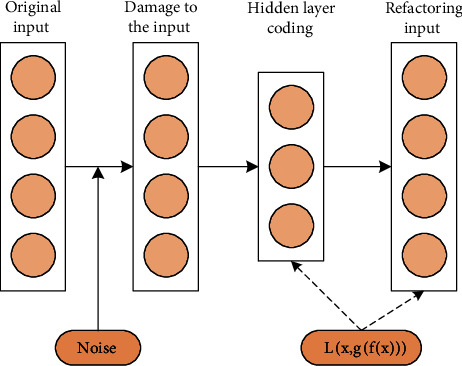
Noise reduction encoder algorithm structure.

In the actual situation, the main considerations are the operating environment of the management system and the network environment. This requires that the extracted function value must have good robustness, and tries to obtain the absorbed sound in different situations and environments corresponding to the function value. It is necessary to improve the original auto-encoder structure and add noise to generate noise reduction codes, as shown in Figure 2.

This will create a new hidden layer code for input data and information. When decoding, the decoder can estimate the initial information that has been interfered from the information without noise interference, and send most of the initial information to reduce the impact of these noises. After multiple in-depth training, the function value improves the robustness.

For noise reduction coding calculation, the general algorithm is: consider the initial input data *x*, add noise to the input data, and obtain new input data. When coding, the characteristic formula obtained is

(4) $$\begin{array}{c} h=f\left(\tilde{x}\right)=s_{f}\left(w\tilde{x}+p\right)\text{.} \end{array}$$

The output obtained during decoding is

(5) $$\begin{array}{c} y=g\left(h\right)=s_{g}\left(\tilde{x}h+q\right)\text{.} \end{array}$$

The final loss function of Ender is

(6) $$\begin{array}{c} J_{DAE}\left(\theta \right)=\underset{x\in s}{\sum }L\left(x,g\left(f\left(\tilde{x}\right)\right)\right)\text{.} \end{array}$$

### 3.2. Construction and Training of Acoustic Model Based on Deep Learning

**Figure 3:**
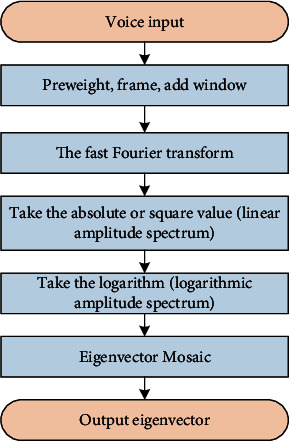
Feature extraction flowchart of spectrogram.

When the spectrogram function is decomposed, the Fourier transform is only performed on each frame signal within a short time after the frame is divided, and the spectrum is obtained through the window. Then square the modulus spectrum to get the energy spectrum, and then take the logarithm to get the logarithmic energy spectrum of each frame. The logarithmic conversion makes the low-amplitude component higher than the high-amplitude component, so you can see the signal period hidden in the low-amplitude noise. Finally, the logarithmic spectrum calculated for each frame is rotated and decomposed in time to obtain the final characteristic sequence spectrum.  Figure 3 is a calculation diagram of the parameters of the spectrogram function.

In the Fourier transformation, the transformation of a nonperiodic continuous time signal *x*(*t*) is

(7) $$\begin{array}{c} X\left(\omega \right)=\overset{\infty }{\underset{0}{\int }}x\left(t\right)e^{-j\omega t}dt\text{.} \end{array}$$

Given a discrete signal of finite length *x*(*n*), *n* = 0,1,…, *n* − 1, the discrete Fourier transform is

(8) $$\begin{array}{c} X\left(k\right)=\overset{N-1}{\underset{n=0}{\sum }}x\left(n\right)W_{N}^{kn},K=0,1N-1,W_{N}=e^{-j2\pi /N}\text{.} \end{array}$$

In the basic acoustic model of this article, the middle layer 3 adopts the pool layer module. Each convolutional pool layer module is two layers with a folding layer + 1 layer pool layer. The fully connected layer adopts two hidden layers, and the number of modules constructed is 1,425, and the size of the rear probability matrix of 200 × 1425 is transmitted via Softmax. The specific structural parameters are shown in Table 1.

### 3.3. Optimized Design of Deep Learning Speech Enhancement Baseline System

**Figure 4:**
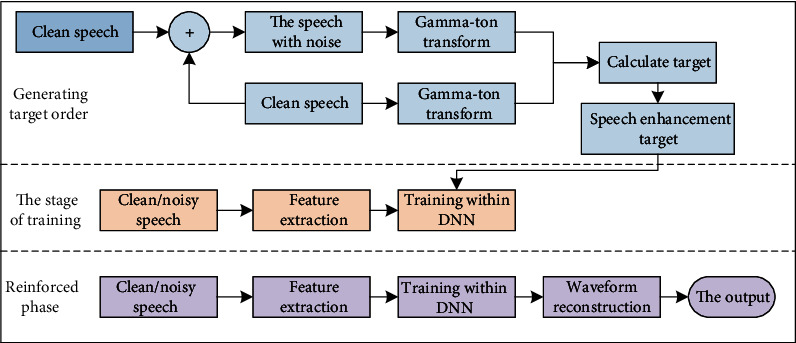
The general flow chart of the deep neural network speech enhancement method.

The deep neural network has strong nonlinear adaptability and can obtain the nonlinear mapping relationship between noisy speech and speech. The deep neural network improvement algorithm includes two processes. The specific process is shown in Figure 4.

Before subjective evaluation, testers must first understand the scoring criteria and then give them more audit training so that the testers can evaluate the sound quality more accurately. Subjective and objective scoring uses the average sentence core scale method. The MOS score is mainly divided into five levels to evaluate the quality of the sound. The score reflects the process of voice quality. See Table 2 for details.

The normalization method in this article is BN. It can converge quickly and improve the versatility of the network. Because the use of BN may interfere with the test and training data, the batch test data is different, which helps to improve the accuracy of the model. BN needs to learn these parameters when normalizing the *γ* and *β* parameters, and transform and construct the normalized data to break the learned function distribution. Assuming that the input of the small batch data is B and the parameters to be learned are *γ*, *β*, then the calculation of BN is: the average calculation formula:

(9) $$\begin{array}{c} \mu_{Β}=\frac{1}{m}\overset{m}{\underset{i=1}{\sum }}x_{i}\text{.} \end{array}$$

Variance calculation formula:

(10) $$\begin{array}{c} \sigma_{B}^{2}=\frac{1}{m}\overset{m}{\underset{i=1}{\sum }}{\left(x_{i}-\mu_{Β}\right)}^{2}\text{.} \end{array}$$

Standardized calculation formula:

(11) $$\begin{array}{c} {\hat{x}}_{\iota }=\frac{x_{i}-\mu_{Β}}{\sqrt{\sigma_{Β}^{2}+\in }}\text{.} \end{array}$$

Modification formula:

(12) $$\begin{array}{c} y_{i}=\gamma {\hat{x}}_{\iota }+\beta \equiv BN_{\gamma \beta }\left(x_{i}\right)\text{.} \end{array}$$

The entrance of the previous layer is directly transmitted to the next layer. The expression output from each remaining module is

(13) $$\begin{array}{c} H\left(x\right)=F\left(x\right)+x\text{.} \end{array}$$

### 3.4. Experimental Results and Analysis

As the speed and volume of the training set body change, the signal length of the training body changes accordingly. The method described in this paper is applied to the regeneration of the adjusted GMM-HMM system of the velocity distortion data and the adjusted GMM-HMM system of the volume adjustment data, for DFSMN acoustic model training based on the speech enhancement data. First, the experiment researched the influence of the speed distortion-based speech data amplification method on the acoustic DFSMN model recognition. The experimental conditions and results are shown in Table 3:

Secondly, the experiment researched the effect of speech enhancement data on DFSMN acoustic model recognition; see Table 4 for details.

Based on the abovementioned experimental results, the discriminant training method based on the sMBR criterion, the treble adaptive method based on the discriminant vector function, and the voice data amplification method based on speed distortion are further combined. The experimental results of these three methods are shown in Table 5.

A speech recognition system using DFSMN and TDNN acoustic models can recognize and transcribe experimental datasets without manual annotations. Among them, DFSMN chose the best model so far, and its vocabulary on the test set is 20.34%. The word error rate on the TDNN test set is 29.88%, so the DFSMN recognition result is regarded as a text label, and the word error rate of each voice recognition result in the dataset is calculated and determined by TDNN, as shown in Table 6.

Table 6 shows that the distribution of word error rate in each time interval is more balanced. Because of the insertion problem in speech recognition, the vocabulary error rate of a single voice may exceed 100%. Therefore, we selected audio tapes with vocabulary frequencies of 0–10%, 10%–20%, and 20%–30% as the extended dataset, used the recognition results of these audio tapes on DFSMN as text annotations, and added them to the existing speech training package, as shown in Table 7.

### 3.5. Fine-Tuning and Optimization of Deep Learning Speech System

When the RBM model is completed, a classifier is added on top of the pattern classification. Compared with logistic regression, only two types of nonlinear classification can be performed. The Softmax method is used to extend logistic regression to complete multiple classifications. They are mutually exclusive, that is, two classifications cannot be occupied by a sample at the same time to meet the experimental requirements. The specific method is given below.

In order to minimize the difference between the actual output and the expected output of the model, the fault uses cross-human correction.

(14) $$\begin{array}{c} H\left(r,S\right)=-\overset{d}{\underset{i}{\sum }}\left(r_{i}\log S_{i}+\left(1-r_{i}\right)\log\;\left(1-S_{i}\right)\right)\text{.} \end{array}$$

Train network parameters *i* by minimizing failures.

(15) $$\begin{array}{c} \theta^{*}={argmin}_{\theta }H\left(r,S\right)\text{.} \end{array}$$

Finally find the child thread:

(16) $$\begin{array}{c} \frac{\partial H\left(r,S\right)}{\partial \theta }=-\overset{d}{\underset{i=1}{\sum }}\left(r_{i},S_{i}\right)\frac{\partial g_{i}}{\partial \theta }\text{.} \end{array}$$

The partial derivatives of W and *b* are

(17) $$\begin{array}{c} \frac{\partial H\left(r,S\right)}{\partial W}={\left(S-r\right)}^{T}X\text{,}\\ \frac{\partial H\left(r,S\right)}{\partial b}=S-r\text{.} \end{array}$$

The process of using the gradient reduction method to update the weights is

(18) $$\begin{array}{c} W^{\prime }=W-\eta \left({\left(S-r\right)}^{T}X+\lambda W\right)\text{,}\\ b^{\prime }=b-\eta \left(S-r+\lambda b\right)\text{.} \end{array}$$

**Figure 5:**
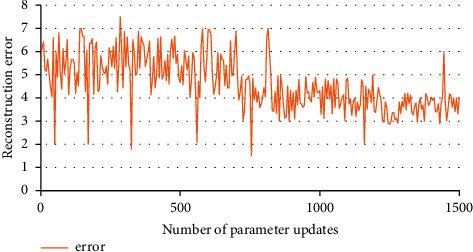
RBM reconstruction performance.

Taking the time-distorted Mel cepstral coefficient and the first-order differential mixing parameter of Mel cepstral coefficient as input data, one can construct classification in the output for identification. If the amount of sample data is small and the number of hidden layers is large, the experimental effect is better, but the relative time spent increases sharply. The experiment process improves the network model according to the set rules and improves the learning efficiency of the model. RBM reconstruction performance is show in Figure 5.

## 4. Research on App Design and Communication in Political Education

### 4.1. Design Principles and Structure Analysis of the Ideological and Political Education App

#### 4.1.1. The Principle of Scalability

With the rapid development of Internet technology and the advancement of society, students' requirements for learning platforms will also change accordingly. Therefore, when developing and designing a platform, not only must it meet the requirements of primary school students, but it must also leave opportunities for future development and improvement. This leaves room for development in subsequent content and function expansion.

#### 4.1.2. Principle of Feasibility

This principle needs to fundamentally complete the research and development of the main functions under the actual conditions of the current software and hardware and the operation of student mobile terminals.

#### 4.1.3. Safety Principles

First, when developing the platform, it is necessary to fully consider the information security situation, suspend user permissions, and protect user privacy and platform security. Second, the platform should try to avoid abusing users, and the interface should be concise and clear to reduce the number of abused users; the information prompt function of the operation should be set so that the user clearly knows the result of this operation.

#### 4.1.4. Principle of Good Interactivity

Good interactivity is very important for the development and design of this application. In this process, the student must be the center. First of all, we must meet the needs of students. We must use the simplest and most practical learning results to achieve the most optimized goal.

**Figure 6:**
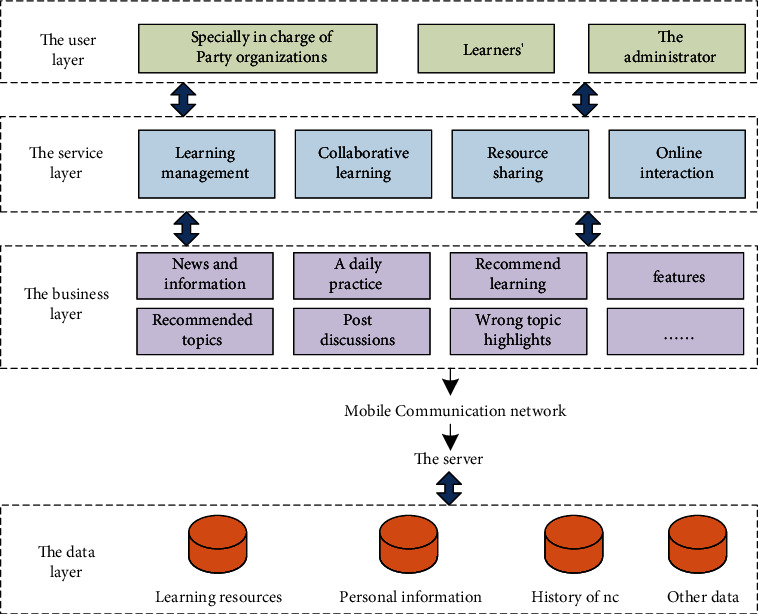
App overall architecture.

The ideological and political education app combines the characteristics of the mobile learning environment, the design concept, and development principles of the app and is a new device for carrying out ideological and political education activities and a new way of mobile learning. The details are shown in Figure 6.

### 4.2. Database Design of Ideological and Political Education App

Table 8 is a detailed description of various learning resource modules.

Sound performance, complete functions, and fast operation are closely related to the database. Therefore, comprehensive consideration must be given to database design. Through the analysis of the application of the ideological and political education app, this article points out that the units of the database design mainly include elementary school students, news information, topic discussions, etc.

#### 4.2.1. User Information Table

The structure of the specific user information table is shown in Table 9.

#### 4.2.2. News Information Table

The detailed content of the specific news information table is shown in Table 10.

### 4.3. Performance Test and Guarantee Design of Ideological and Political Education App

When the network is transplanted to the last layer, the neural network compares the predicted situation with the actual situation and calculates the deviation inside. This calculation function is also called the loss function. The commonly used loss function is the cross entropy loss function, and the calculation formula is

(19) $$\begin{array}{c} J\left(W,b,a,y\right)=-y\cdot \ln\;a-\left(1-y\right)\cdot \ln\;\left(1-a\right)\text{.} \end{array}$$

The concept of the gradient formed by the *j*th neuron in the *l*th layer is

(20) $$\begin{array}{c} \delta_{j}^{l}=\frac{\partial C}{\partial Z_{j}^{l}}=\frac{\partial C}{\partial a_{j}^{l}}\cdot \frac{\partial a_{j}^{l}}{\partial Z_{j}^{l}}\text{.} \end{array}$$

*C* is the value of the loss function to calculate the deviation of the last layer of the neural network

(21) $$\begin{array}{c} \delta^{l}=\frac{\partial C}{\partial a^{l}}•\frac{\partial a^{l}}{\partial Z^{l}}\text{.} \end{array}$$

Then continue to calculate the deviation of the previous layer

(22) $$\begin{array}{c} \delta^{l}=\left({\left(w^{l+1}\right)}^{T}\delta^{l+1}\right)•f^{\prime }\left(z^{l}\right)\text{.} \end{array}$$

And the slope of the previous layer

(23) $$\begin{array}{c} \frac{\partial c}{\partial w_{jk}^{l}}=a_{k}^{l-1}\delta_{j}^{l}\text{,}\\ \frac{\partial c}{\partial b_{j}^{l}}=\delta_{j}^{l}\text{.} \end{array}$$

Assuming that the learning speed is set, the learning speed must be multiplied by the weight *w* and the deviation *b* of the variable update. Using the software testing method, package the application in the form of an apk and install it on a suitable machine, and test the main functions of the application, as shown in Table 11.

### 4.4. Analysis of App Communication Strategies in Ideological and Political Education

Usually, people generally pay attention to the information they are interested in. Therefore, we must first take the ideological and political training app as the focus of party building and focus on the timely change and system optimization of the app's content. Therefore, when disseminating and promoting ideological and political education apps, we should focus on the needs of the public, pay attention to customer feedback, choose effective content from the platform, and promote interactive circular information. The application of ideological and political education is very important for the rapid dissemination of information. In the Internet era, the public has access to a huge amount of information, and attention has become less important in the media. The speed and area of information transmission will have the greatest effect on the success or failure of evaluation and management.

Although the content of the ideological and political education application is different from other applications that people know, it is also a mobile terminal application that should pay great attention to user experience, system innovation, and optimization of functions and uses. Because the application is suitable for mobile terminal devices, the environment used by the application is mainly a mobile environment. Therefore, the convenience of product operation and user comfort have become the main criteria for evaluating the experience of application users. Therefore, the primary goal of optimizing the process of ideological and political education app user experience is to be direct, simple, fast, comfortable, and easy to understand and give people a user-friendly and easy-to-use experience.

## 5. Conclusion

Different types of applications and diverse scenarios enable users to experience different personal experiences. This paper examines the use of application software for ideological and political theory courses in colleges and universities and the application of application software for ideological courses and analyzes the use of apps in the management of ideological and political theory courses in colleges and universities. Including the development and design of ideological and political theory applications, combining deep learning and CTC algorithms to build acoustic models, using server-client interaction, designing an ideological and political theory course app based on speech recognition and deep learning, and forming an offline speech recognition system software platform, the app provides a certain reference for improving the skills of teaching managers.

## Figures and Tables

**Table 1:** CTC-CNN baseline acoustic model parameters.

Network layer	Parameter
InputLyer	300 dimensional harmony
conv2d_Lyer1	33 convolution channels, convolution kernel 4 × 4, step size 3 × 3, activation function
conv2d_Lyer2	33 convolution channels, convolution kernel 4 × 4, step size 3 × 3, activation function
max-poolng2d	Maximum pooling 3 × 3
conv2d_Lyer3	65 convolution channels, convolution kernel 4 × 4, step size 3 × 3, activation function
conv2d_Lyer4	65 convolution channels, convolution kernel 4 × 4, step size 3 × 3, activation function
max-poolng2d	Maximum pooling 3 × 3
conv2d_Lyer5	129 convolution channels, convolution kernel 4 × 4, step size 3 × 3, activation function
conv2d_Lyer6	129 convolution channels, convolution kernel 4 × 4, step size 3 × 3, activation function
max-poolng2d	Maximum pooling 3 × 3
Reshap	Feature map transformation output 300 × 3300
FC_Lyer1	Number of neurons 129, activation function
FC_Lyer2	The number of neurons is 1425, the activation function
Softmx	Activation output matrix, the dimension is 300 × 1425
CTC	Probability matrix, the length of the sonogram, the length of the label sequence

**Table 2:** MOS scoring and related application description.

MOS score	Quality level	Distortion level
5	Great	Not aware
4	Better	Slightly aware
3	General	Aware, but lighter
2	Poor	Clearly aware
1	Difference	Very obvious

**Table 3:** Comparison of experimental results based on velocity disturbance.

Model	WER (%)	SER (%}
Baselne	25.95	78.73
SP09	22.68	68.08
SP11	22.81	68.32
SPe-BAND	22.58	67.98
SP09 + SP11	21.49	67.65
SP-RAND2	21.73	67.74

**Table 4:** Comparison of experimental results based on volume gain adjustment.

Model	WER (%)	SER (%)
baselne	25.95	78.73
VL05	22.85	68.58
VL15	22.83	68.53
VLe-RAND	22.88	68.64
VL05 + VL15	23.98	69.75
VL-RAND2	22.94	68.94

**Table 5:** Comparison of the results of the fusion of the three optimization methods.

Model	WER (%)	SER (%}
baselne	25.95	78.73
i-vctor + sMBR	23.18	69.13
SP09 + SP11	21.49	67.65
i-vector + sMBR + SP09+SP11	20.34	63.88

**Table 6:** Recognition result distribution statistics.

WER (%) interval	Duration (hours)	Proportion (%)
0–10	21.4	10.66
10–20	20.2	10.06
20–30	21.7	10.81
30–40	24.6	12.26
40–50	26.3	13.11
50–60	18.5	9.21
60–70	17.6	8.76
70–80	16.8	8.36
80–90	14.5	7.21
90–100	11.3	5.61
≥100	8.2	4.06

**Table 7:** Automatically annotated speech data augmentation experiment.

Model	WER (%)	SER (%}
Baselne	25.95	78.73
DFSMN (0–10)	21.88	66.68
DFSMN (10–20)	22.17	67.89
DFSMN (20–30)	23.96	70.45

**Table 8:** Organization and classification design of learning resources.

Learning capital	Functional module	Text, pictures, charts	Features
Study plan	Study plan	Text, image, audio and video, etc	Understanding the learning plan

Learning resource library	Daily practice	Text, image, page	Provide various forms of learning content to promote students' independent learning
	News report		
	Study item		
	Column content		

Learning activity library	Activity theme	Text, page	Provide topic discussions to help students work together to solve problems
	Main content		
	Start a discussion		

Learning evaluation library	Daily practice	Text, page, monitoring	Assess student learning effectiveness
	Quiz		

Learning monitoring library	Practice daily monitoring of learning	Text, image, table	Monitor student learning
	Self-monitoring		
	Student monitoring		

**Table 9:** User information form.

Field content	Type of data	Content description
Id	inte(10)	Self-growing primary key
Usrname	varehr(100)	User name
Pasword	varchr(32)	User password
Headmg	varchr(255)	User image
Sex	inte(1)	Gender
Stats	tinynt(1)	User status
reg_tme	inte(10)	Log in time
last_logn_time	inte(10)	Log in time
last_logn_ip	varchr(15)	Login address
Tokn	varchr(100)	Instruction board
Token tme	inte(11)	Expire date
study_pan	varchr(255)	Study plan
Phon	varchr(20)	Contact number
Long _tme	inte(10)	Cumulative study time

**Table 10:** News information sheet.

Field content	Type of data	Allow to be empty	Content description
Id	inte(11)	Not allowed	Increase primary key
User_id	inte(11)	Allow	User address
News_id	inte(11)	Allow	News category address
Title	varehr(255)	Allow	Topic
Url	varchr(100)	Allow	Connection
Status	varchr(255)	Allow	Subscribe

**Table 11:** Login registered test case table.

Use case number	Side trial example	Expected results	Test result
1	Click client	The login interface appears	Accord with
2	Enter the registration page	Jump to the registration interface	Accord with
3	Enter the phone number to get the verification code	Sending text verify code	Accord with
4	Write verification code	Enter the verification code correctly and jump to the registration interface	Accord with
5	Supplement information and submit	Successful registration appears, enter the main interface	Accord with
6	Enter the system again	Go directly to the main interface	Accord with
7	Click the back button	Back to the previous level	Accord with

## Data Availability

The data used to support the findings of this study are available from the corresponding author upon request.
